# Facile synthesis and nanoscale features of a nanostructured nordihydroguaiaretic acid analog for therapeutic applications

**DOI:** 10.1186/s12951-020-00628-z

**Published:** 2020-05-14

**Authors:** Geraldine Sandana Mala John, Veena Kumari Vuttaradhi, Satoru Takeuchi, Ravi Shankar Pitani, Ganesh Venkatraman, Suresh Kumar Rayala

**Affiliations:** 1 Department of Biotechnology, Indian Institute of Technology, Madras, Chennai, Tamilanadu 600036 India; 2 Factory of Takeuchi Nenshi, TAKENEN, 85NE Takamatsu, Kahoku, Ishikawa 929-1215 Japan; 3 Department of Community Medicine, Sri Ramachandra Institute of Higher Education & Research, Porur, Chennai, Tamilnadu 600116 India; 4 Department of Human Genetics, Sri Ramachandra Institute of Higher Education & Research, & Sri Ramachandra Center for Biomedical Nanotechnology, Porur, Chennai, Tamilnadu 600116 India

**Keywords:** Acetyl Nordihydroguaiaretic acid, Atomic force microscopy, Differential scanning calorimetry, FT-IR spectroscopy, Polymeric nanospheres, X-ray Diffraction technique

## Abstract

**Background:**

Nordihydroguaiaretic acid (NDGA) is a plant lignan obtained from creosote bush, known to possess anti-oxidant, anti-cancer and anti-viral activities and is being used in traditional medicine. However, toxicity studies indicated liver and kidney damage despite its immense medicinal properties. There has been a recent increase of curiosity in the chemical synthesis of NDGA derivatives for therapeutic applications. NDGA derivatives have been developed as better alternatives to NDGA and for targeted delivery to the site of tissue by chemical derivatives. In this regard, an analog of NDGA, Acetyl NDGA (Ac-NDGA), has been synthesized based on a previous procedure and formulated as a nanostructured complex with Polycaprolactone/Polyethylene glycol polymer matrices, by o/w solvent evaporation method.

**Results:**

The drug-incorporated polymeric nanospheres exhibited a drug load of 10.0 ± 0.5 µg drug per mg of nanospheres in acetonitrile solvent with 49.95 ± 10% encapsulation efficiency and 33–41% drug loading capacity with different batches of nanospheres preparation. The in vitro drug release characteristics indicated 82 ± 0.25% drug release at 6 h in methanol. Further, the nanospheres have been characterized extensively to evaluate their suitability for therapeutic delivery.

**Conclusions:**

The present studies indicate a new and efficient formulation of the nanostructured AcNDGA with good therapeutic potential.
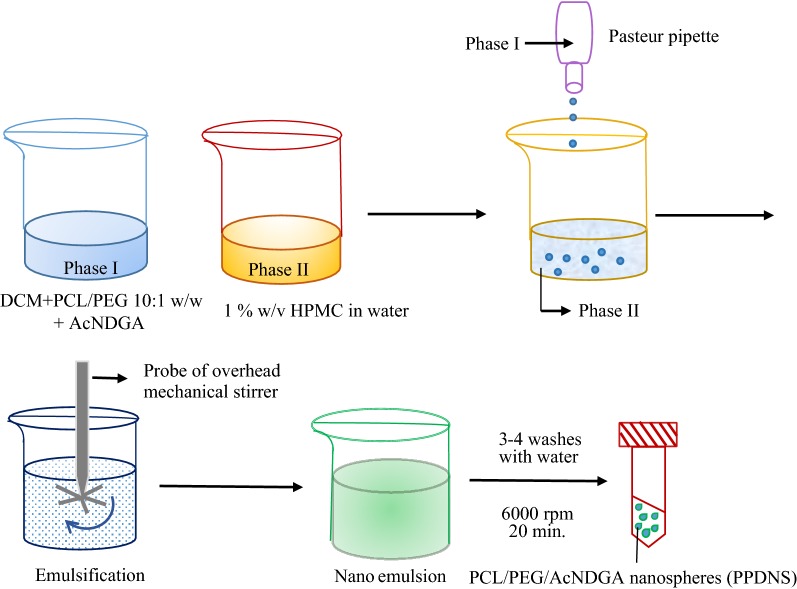

## Introduction

Nordihydroguaiaretic acid (NDGA) is obtained from creosote bush, *Larrea tridentata*, commonly found in desert regions of North America and Mexico. NDGA is a plant lignan and possesses several important biological activities. NDGA is a lipoxygenase inhibitor and is known to possess anti-oxidant, anti-cancer and anti-viral activities and is used in traditional medicine. It is commonly referred as ‘chapparal’ and consumed as tea to treat several medical ailments [1]. However, its prolonged consumption leads to hepatotoxicity [1–3] and formation of renal cysts [4] as reported from in vivo and in vitro experimental studies. It was marketed as Actinex^®^ as an anti-neoplastic drug in 1992. However, the FDA banned its use in medical ailments in 1996 due to low market demand and few reported cases of side effects [5]. The therapeutic effects of NDGA has been investigated in cancer [6] as a hypoglycemic agent [7], as an anti-inflammatory and analgesic drug in the treatment of arthritis [8] and stomach ulcers [9]. But, there is little awareness of its toxicity effects rarely reported in literature or established in animal models. During the past two decades, chemical synthesis of NDGA analogs have been carried out by several researchers demonstrating the newly acquired properties/therapeutic effects of the derivatized NDGA with different chemical moieties [10–12]. This has been a promising approach to reduce/eliminate the toxicity implications of the native drug, but there is also a need for further investigations of the reduction of toxicity of NDGA analogs in vivo by supported literature.

Nanotechnology has been emerged as a broad scientific discipline encompassing material science, physics, chemistry, medicine, pharmacology and biotechnology as well and has been investigated for about 25–30 years to effect various applications in science and technology. The worldwide market for nanoparticles in biotechnology and pharmaceuticals industry reached nearly 25.0 billion dollars in 2013 and this market is expected to reach $79.8 billion in 2019 [13]. Polymeric nanoparticles are exceptional vehicles for site-directed deliverance of drugs [14–18] and are a proficient platform for drug delivery [19]. Further, drug nanocarriers have been considered as the future of chemotherapy for targeted delivery to tumor tissues [20].

In Nanomedicine, nanotechnology is applied for diagnostic, therapeutic strategies and in regenerative medicine for various biomedical and biomaterial scientific developments. In drug delivery, despite the vast amount of pharmacological drugs that are developed for clinical trials, most of the drugs fail to enter the pharmaceutical market due to inefficiency of drug delivering modules even if the drug has been identified as a potent candidate for treatment of several medical disorders by in vitro and in vivo studies. Therefore, drug discovery is one aspect where several drug candidates are in the pipeline, while, proper targeting of the drugs are of significant concern in recent years. Nanotechnology has been able to be utilized for drug delivery approaches imparting several novel features to the drug delivery device/module to achieve targeted delivery with tissue specificity by passive diffusion or by binding with cell-surface receptors or by endocytotic process within the cells. Thereby, passive or active targeting of the drug to the cells is being accomplished from the nano device [21]. To achieve this, nanoformulations need to possess suitable therapeutic characteristics which enable them to carry the drug to the appropriate tissue site and affect a therapeutic response. Thus, the nanoscale features of the drug formulations are critical for therapeutic delivery, which decide the fate of the drug within the physiological system as well as the drug release properties.

Cancer diagnosis and cancer therapeutics are particularly benefited by the advent of Nanomedicine for bioimaging and drug delivery. Targeting cancer tissues/cells has been the most intriguing tasks of Nanomedicine for several years and also of recent research interests. This holds good for future promises of cancer therapy. The rapid advancements in Nanotechnology/Nanomedicine account for such an increased input of scientific interests in cancer diagnosis and treatment. Nanoparticle-mediated drug delivery in anti-cancer therapy has been investigated up-to-date for better treatment procedures and patient compliance [22–26]. Polymeric nanoparticles have been largely employed for targeted drug delivery due to significant advantages in cancer nano therapy. Nanoparticle-mediated cancer therapies offer: (1) low toxicity due to decreased therapeutic dosage; (2) ability to traverse blood–brain barrier; (3) site-directed therapy by surface-modified chemical derivatives; (4) controlled drug release over a time period; (5) enhanced tumor penetration; (6) use in combination therapies; (7) overcoming drug resistance; (8) increased cellular uptake; (9) increased bioavailability; (10) recognition of cancer cells by ‘memory’ of targeting; (10) permitting design of different forms for various therapeutic requirements; (11) non-interference to healthy cells and; (12) improved drug solubility. These advantages of nanoparticles prompt more research interests to find more solutions to the increasing rates of cancer incidences worldwide.

Considering these aspects, we have standardized the preparation of a polymer-based nanoformulation of Acetyl NDGA (Ac-NDGA) with polycaprolactone (PCL) and Polyethylene glycol (PEG) by an o/w emulsification solvent-evaporation protocol and characterized it extensively by different microscopic and spectroscopic techniques to assess the efficiency of the drug nanoformulation in therapeutic delivery. The drug load and drug release profiles have also been determined to estimate the capacity of the nanoformulation for therapeutic delivery. Further, the cytotoxicity of the nanoformulation has been studied on HepG2, a liver cancer cell line by MTS [3-(4, 5- dimethylthiazol-2-yl)-5-(3-carboxymethoxyphenyl)-2-(4- sulfophenyl)-2 *H*-tetrazolium] assay to evaluate the anti-cancer potential of the drug-loaded nanospheres for drug delivery applications.

## Methods

### Materials and reagents

Nordihydroguaiaretic acid (NDGA), Polycaprolactone (PCL, Av.MW 10,000), Polyethylene glycol (PEG, Av.MW 400) chemicals were obtained from Sigma Chemicals Co. (India). All other chemicals and solvents were of reagent grade.

### Synthesis of tetra-*O*-acetyl nordihydroguaiaretic acid derivative

Acetyl NDGA was synthesized by a chemical procedure as previously described by Plaza et al. [27] with some conditions adapted in our laboratory. NDGA (1.5 g; 4.96 mmol) was taken in a 100 ml screw-capped Erlenmeyer flask and 3.0 ml (6 times of 4.96 mmol NDGA) of acetic anhydride was added to the NDGA in aliquots of 1 ml and gently mixed. About 2.5 µl of conc. H_2_SO_4_ was added to the reaction mixture and shaken by hand until it completely dissolved. The reaction mix was kept overnight at room temperature to ensure complete reaction. About 10 ml of ice was taken in another 100 ml Erlenmeyer flask and the reaction mix was added. A buttery yellow solid was obtained which was filtered in a Buchner funnel under vacuum and washed with distilled water and pressed to remove traces of water. The pressed filter-cake was dissolved in 95% ethanol by heating to 80 °C and kept at room temperature for re-crystallization overnight. The Tetra-*O*-Acetyl NDGA crystals were filtered in a Buchner funnel and dried in a vacuum dessicator overnight until constant weight. The filtrate was kept until further re-crystallization and the crystals were treated similarly and weighed. The percent yield of the tetra-acetyl NDGA derivative (AcNDGA) was calculated. The structure of AcNDGA was assessed by ^1^H-NMR spectroscopy. The elemental composition (C, H) of AcNDGA was also determined by CHN analyzer, Perkin-Elmer CHN 2400. Ac-NDGA was characterized by ATR (Attenuated Total Reflectance) FT-IR for analysis of functional groups. The molecular weight of AcNDGA was determined by ESI–MS to confirm the tetra-acetylated molecular structure. This tetra acetylated derivative (Ac-NDGA) was used for preparation of Nanospheres and characterized.

### Preparation of PCL/PEG/AcNDGA Nanospheres by o/w emulsion method

About 2 g of PCL was dissolved in 20 ml of Dichloromethane and added 178 µl of PEG (10:1 w/w, 0.2 g/density per ml) in a 100 ml screw-capped Erlenmeyer flask, and mixed well. AcNDGA (20 mg) was added to the polymer solution and mixed well. A control set of the polymer formulations without the drug and sonicated for 1 min was also carried out. The polymer solutions were added drop wise to 1% (w/v) Hydroxy Propyl Methyl Cellulose (HPMC) (external aqueous phase) solution in milli-Q water. The resulting emulsion with 1% HPMC was stirred using an overhead mechanical stirrer for 30 min. The nanospheres were collected by centrifugation at 6000 rpm for 20 min. The nanospheres were further washed with milli-Q water three times to remove traces of HPMC and solvent, and vacuum dried to constant weight. The percent yield of the nanospheres was calculated and the nanospheres obtained were designated as PPNS (without drug load) and PPDNS (with drug load).

### Drug loading capacity of PPDNS

About 30 mg of PPDNS was dissolved completely in 3 ml of Acetonitrile and the absorbance at 282 nm was measured using a Nanodrop instrument (ThermoScientific, USA). The amount of the drug was determined from the calibration graph of 0–12.5 mg/3 ml Ac-NDGA in Acetonitrile [28]. The drug loaded in the nanospheres was determined as follows:

$${\text{Drug load }}\left( {\text{mg}} \right) \, = \frac{{{\text{Wt}}.{\text{ of the drug in the sample }} \times {\text{ Wt}}.{\text{ of the nanospheres in the batch preparation}}}}{{{\text{Wt}}.{\text{ of nanospheres taken}}}}$$

$${\text{Encapsulation efficiency }}\left( \% \right) \, = \frac{{{\text{Wt}}.{\text{ of drug in the nanospheres}}}}{{{\text{Wt}}.{\text{ of feeding drug}}}} \times 100$$

$${\text{Drug Loading capacity }}\left( \% \right) \, = \frac{{{\text{Wt}}.{\text{ of drug in the nanospheres}}}}{{{\text{Wt}}.{\text{ of nanospheres taken}}}} \times 100$$

### In vitro drug release of PPDNS

About 50 mg of PPDNS was suspended in 2 ml of methanol in 5 ml eppendorffs and mixed in a rotaspin for 15 min, 30 min, 1, 2, 3, 4, 6 h at room temperature and for 8, 10 and 24 h at 4 °C. At the specified time intervals, the samples were centrifuged, the volume of the supernatant was noted, about 0.3 ml aliquots of the supernatant was taken in a 96-well plate in triplicates and the absorbance of the samples along with methanol blank was read at 284 nm [28]. The amount of Ac-NDGA released was determined from the calibration graph of 0–300 µg/ml Ac-NDGA in methanol.

### Characterization of PPDNS

PPDNS were characterized by Dynamic Light Scattering (DLS), Attenuated Total Reflectance (ATR) FT-IR, Scanning Electron microscopy (HR-SEM), Powder XRD spectroscopy, Atomic Force Microscopy (AFM), Electrospray Ionization Mass spectrometry (ESI–MS), Thermo Gravimetric Analysis (TGA) and Differential Scanning Calorimetry (DSC) techniques.

- Dynamic Light Scattering Measurements
  About 5 mg of PPNS and PPDNS were suspended in 3 ml of milliQ water and dispersed in a sonicator for 1 min at 60% amplitude and analyzed for particle size distribution and zeta potential using a Microtrac instrument with cumulative values of 3 scans for each sample. The particle size distribution in nanometer scale and the zeta-potential were determined with Microtrac FLEX software v. 10.6.2.
- Attenuated Total Reflectance-Fourier Transform-Infra Red Spectroscopy
  Powder sample of PPDNS was applied to a Bruker ATR-FTIR spectrometer without any sample preparation and analyzed between IR spectral range of 600–3500 cm^−1^ wave number in % transmittance mode. The spectral values were recorded for each band of the spectrum and compared with standard values of IR spectrum obtained with known functional groups of Acetyl NDGA, PEG and PCL.
- Powder X-ray diffraction spectroscopy
  About 10 mg of PPDNS was analyzed in a Powder X-ray diffraction spectrometer (DS Advance, Bruker) and the data was plotted in OriginPro software v.8.5.
- Electrospray Ionization (ESI)-Mass Spectrometry
  About 10 mg of PPNS and PPDNS dissolved in 1 ml of dichloromethane was analyzed for molecular mass in a Q-TOF ESI-Mass Spectrometer.
- Atomic Force Microscopy (AFM) analysis
  About 3 mg of PPDNS was sonicated with 100 µl of Milli-Q water in a probe-type sonicator. The dispersed sample was placed on a glass slide and allowed to air-dry at room temperature for 1 h. The air-dried sample was immediately analyzed in an Atomic Force Microscope (Model XE-100, Park Systems) by semi-contact mode scanning. Images of 2D and 3D surface scan at nanoscale and the mean size parameters were obtained.
- High Resolution-Scanning Electron Microscopy/EDX
  PPDNS was placed on a stub and sputtered with gold and analysed by a HR-Scanning electron microscope FEI Quanta 200F (Netherlands) and images were taken. EDX analysis was also carried out to determine the chemical composition of the nanospheres.
- Thermogravimetric analysis
  Powder sample of PPDNS was analyzed for thermal degradation in a Perkin-Elmer TGA7, Q500 Hi-Res TGA (TA instruments) and the transition curve was obtained by heating the sample from 30 to 930 °C at a constant heating rate of 20 °C/min.
- Differential Scanning Calorimetry analysis
  DSC analysis of PPDNS was carried out in a Perkin-Elmer DSC7 Q200 MDSC Analyser (TA instruments) between 10 and 250 °C at a constant heating rate of 20 °C/min and the DSC thermograms were obtained.

### Cell line, culture media and maintenance of cell line

A liver cancer cell line HepG2 was obtained as gift from Sri Ramachandra Medical College and Research Centre, Chennai. The cells were kept frozen in liquid N_2_ until use. DMEM medium-HG (ThermoScientific, USA) was supplemented with 10% Fetal bovine serum (FBS), 1% antibiotic solution of Penicillin/Streptomycin with Amphotericin or Nystatin, 3 ml/l flucanazole solution, 200 mg/l Taxim and was used for the culture of HepG2 cell line. HepG2 cells were maintained in DMEM-HG media at 37 °C in a 5% CO_2_ incubator during the experiments. All procedures were carried out under sterile conditions in a BSL II laminar flow chamber.

### Cytotoxicity analysis

HepG2 cells containing approximately 11.0 × 10^4^ cells/ml were seeded in 96-well plates and incubated in a 37 °C CO_2_ incubator for 24 h. The free drug (AcNDGA) and the drug-loaded nanospheres in culture medium were added at appropriate concentrations in 100 µl/well after 24 h. Cells without drug treatment were used as control. After 24 h and 48 h of drug treatment, 25 µl of MTS reagent (Promega, India) was added to each well and incubated in a 37 °C CO_2_ incubator for 2 h. Absorbance of the samples were then measured at 490 nm using a microplate reader (Molecular Devices, San Jose, USA) and the percentage (%) inhibition was calculated according to formula (1). A MTS control was also used to deduct the absorbance by MTS from the absorbance of the samples.

1 $$\% {\text{ inhibition }} = \frac{{ 100 \, {-} \left( {\text{OD of the individual test group}} \right)}}{{({\text{Mean OD of the control}}}} \times 100$$

The percentage inhibition vs drug concentrations were represented as a box plot to compare the broad range of all groups using SPSS software v16.0.

### Statistical analysis

The data was analyzed and the significance was evaluated by One-way Analysis of Variance (ANOVA) using SPSS software v16.0. The data was presented as mean ± SEM and the *P* value < 0.05 was regarded as significant.

## Results and discussion

NDGA has long been used traditionally as anti-cancer drugs. However, its toxicity to liver cells has prompted recent research to use NDGA analogs with similar anti-cancer activity but lacking toxicity. Hence, this work was carried out to chemically synthesize an efficient NDGA analog and to assess its characteristics for therapeutic delivery. Nanoparticle-based drug delivery systems can improve the overall pharmacological properties of several drug candidates as they can easily traverse the cell membrane and diffuse within the cell matrix. The nanoscale features of the nanoparticles such as size, surface area, improved solubility and multi-functionality allow targeted drug delivery over a sustained period. Controlled release properties of nanoparticles offer lower drug concentrations to be administered systemically or at the target site thereby preventing toxicity due to excess drug accumulation. The size and surface charge of nanoparticles are critical for cellular uptake in tissues/bloodstream. Nanoparticles are excellent candidates for drug delivery applications and are capable of delivering any type of drugs namely hydrophilic or hydrophobic, biological macromolecules including proteins and even vaccines. Nanoparticles have significant advantages as compared with microparticles developed earlier due to size limits of the microparticles that can only remain in Peyer’s Patch while nanoparticles can be systemically distributed. Further, nanoparticles are suited for intravenous administration due to their ability to enter into blood capillaries as small as 5–6 µm diameters [29]. Nanoparticles may be prepared in different forms such as nanospheres, nanofilms, nanofibers, gels and other physical forms. Polymers are best suited drug delivery carriers and have a long history of use as preferred drug vehicles [19]. Several polymers are used as nanocarriers which maybe natural polymers or synthetic polymers. Synthetic polymers such as Polycaprolactone (PCL) gained considerable interest since 1970s and 1980s and was almost forgotten for two decades. In recent years, PCL has been used in several biomedical applications in drug delivery, tissue engineering, in implants and devices owing to its biodegradability, biocompatibility, low immunogenicity and little or no antigenicity [30]. Polymeric nanoparticles are in the order of 1–1000 nm size range and are well-suited for controlled delivery. The widespread method used for the preparation of solid polymeric nanoparticles is the emulsification-solvent evaporation technique, which can efficiently formulate hydrophobic drugs in a nanostructured complex, rather than hydrophilic drugs. Moreover, surface modifications of the polymer matrix can promote targeted delivery of the drug candidates. Hence, the AcNDGA has been formulated with PCL/PEG polymeric matrices as drug-loaded nanospheres and extensively characterized by various spectroscopic and microscopic techniques and assessed for drug loading capacity and drug release properties, in order to evaluate the nanostructured drug complex for efficient therapeutic delivery. The elemental composition of the synthesized AcNDGA was C-66.45%; H-4.24%.

### Structural characterization of AcNDGA

**Fig. 1:**
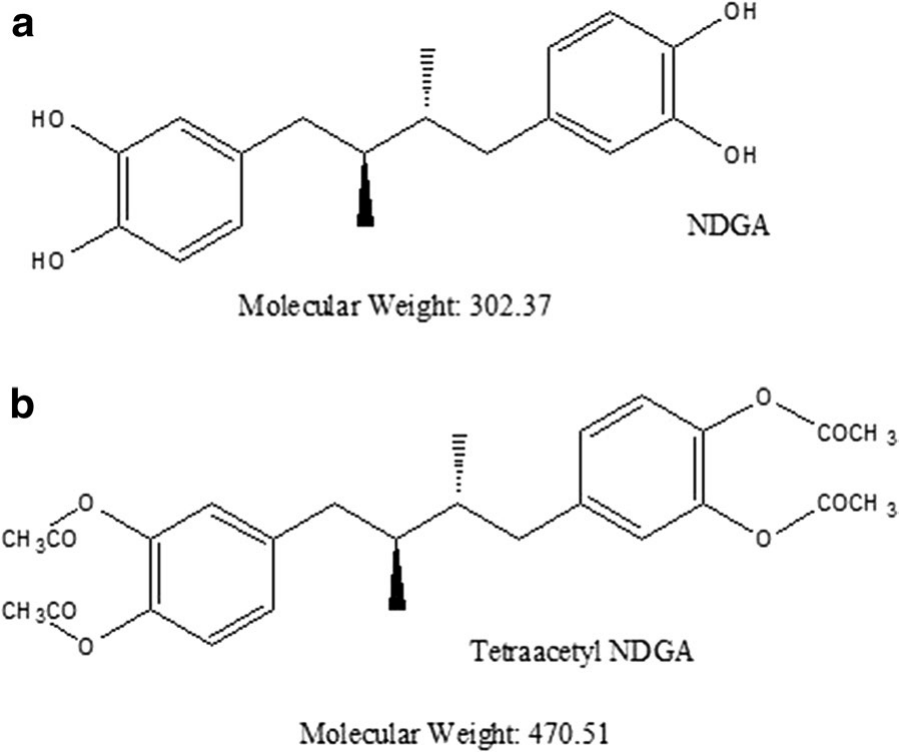
Chemical structures of NDGA (**a**) and Ac-NDGA (**b**)

**Fig. 2:**
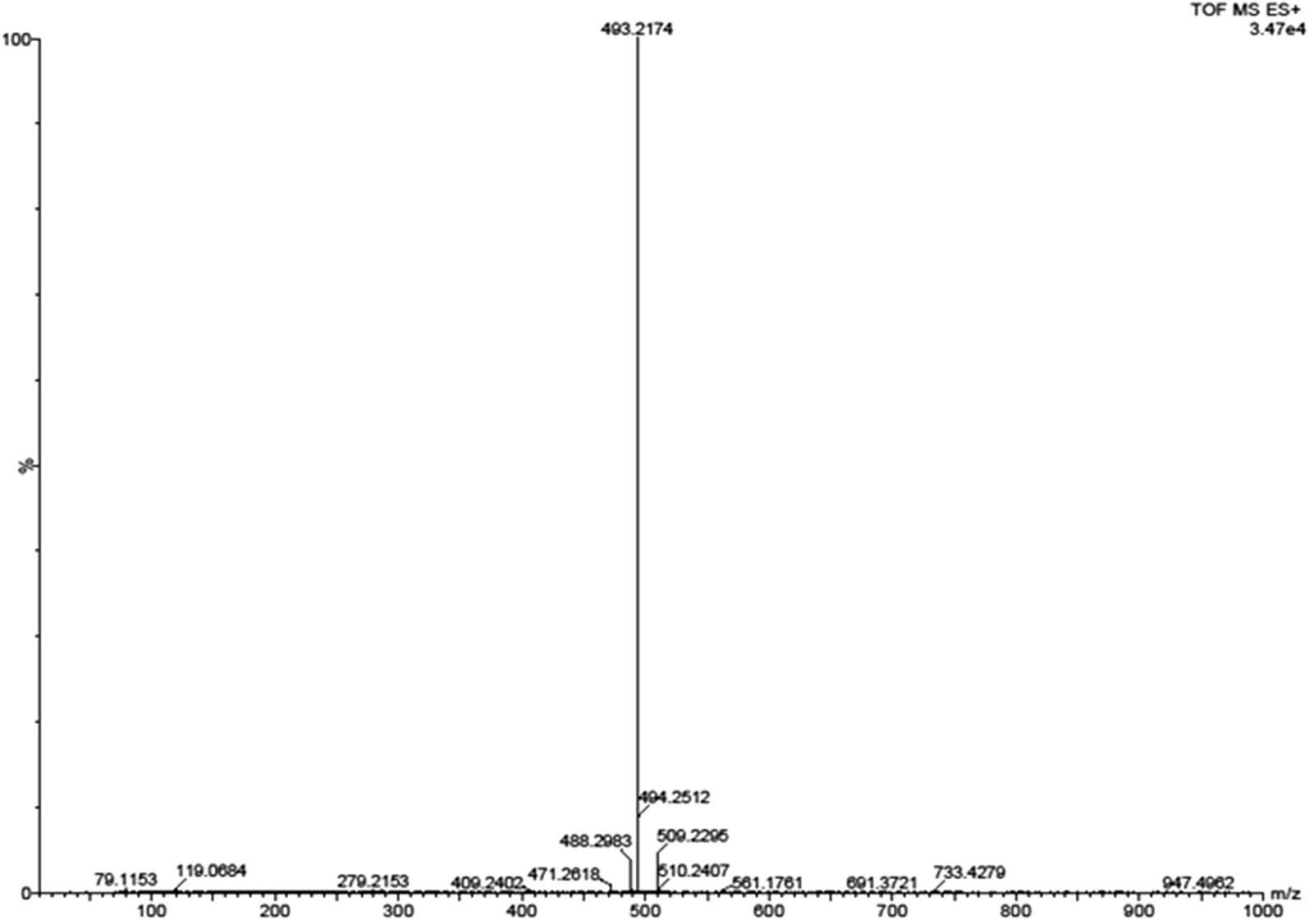
ESI-MS spectrum of Ac-NDGA analysed in +ve ion mode. MW 493.21 (obs)**Calculated MW of Ac-NDGA = 470.52

The compound was characterized further by ^1^H-NMR, ESI–MS and FT-IR to confirm its structure and chemical moieties. The chemical structure of AcNDGA (Fig. 1) has been assessed by ^1^H-NMR spectroscopy. The signals in the NMR spectrum corresponded well with those of theoretically calculated functional groups of AcNDGA (data not shown). The molecular mass of AcNDGA was determined by positive ion mode ESI–MS. The mass spectrum obtained showed a single peak of AcNDGA with an observed mass of 493.21 as compared to the calculated mass of 470.52 due to the protonation of the compound while obtaining the mass spectrum (Fig. 2). The synthesized AcNDGA was characterized for functional groups by ATR FT-IR. The IR spectrum confirmed the presence of functional groups of the compound with similar bond stretches. The alkane C–H stretch was strong at 2970–2929 cm^−1^ and the acid –OH stretch was broad and strong at 2870 cm^−1^. The carbonyl C=O stretch was most intense and strong as well as the ester C=O stretch at 1756–1801 cm^−1^. The 1500–400 cm^−1^ is the characteristic fingerprint region which has unique patterns in nearly all molecules. This region showed single bond stretches and wide variety of bending vibrations (data not shown).

### Preparation of AcNDGA-loaded PCL/PEG nanospheres (PPDNS)

The synthesized AcNDGA has been ascertained for its molecular structure and therefore prepared as nanospheres with PCL and PEG, and characterized. In recent years, focus has been largely on drug-incorporated nanoparticles for efficient drug delivery in a sustained/controlled release manner more than the free drug candidates. This is due to toxicity in excess administration, poor solubility of hydrophobic drugs and systemic circulation without reaching the targeted organ/tissue/cells of the native drugs. Hence, it has been attempted to formulate a nanostructured drug complex with polymers such as PCL and PEG. An advantage of using a dual polymer system is to compromise on the stability of the amphiphilic drug-polymer complex. PCL is a standard synthetic polymer with excellent biodegradability and biocompatibility and is a hydrophobic polymer. PEG is a synthetic hydrophilic polymer and manipulation of the polymer ratios can increase/decrease their solubility patterns. Choice of PEG in this study is due to its low toxicity and use as excipient in pharmaceutical formulations. PEG 400 is of pharmaceutical quality. A 10:1 (w/w) ratio of PCL: PEG was used for preparation of drug-incorporated nanospheres by an o/w emulsification-solvent evaporation method. This method is less cumbersome and does not require cost-intensive equipment. The solvent phase (dichloromethane) contained PCL and PEG polymers w/o the drug. The solvent phase was then added drop wise to the external aqueous phase containing hydroxymethyl propyl cellulose. The resulting emulsion was stirred in a mechanical stirrer for about 30 min and the nanoparticles were washed in milli-Q water to remove excess external phase, and dried under vacuum. This method has been employed as a facile procedure for the preparation of nanospheres in our laboratory using an overhead mechanical stirrer with rapid formation of nanospheres in about 25–30 min stirring, while with lab magnetic stirrers with a maximum of 1000 rpm, overnight stirring is required and have resulted in formation of microspheres. We have developed this protocol based on available literature [31–34] and have avoided freeze drying of nanospheres [31, 32] which caused disruption of the surface of nanospheres as observed in Scanning Electron Microscopy with extensive porosity and drug burst release in methanol. Vacuum drying at room temperature was employed to overcome this effect and we did not want to use lyoprotectant such as mannitol [32] during lyophilization procedure as removal of the lyoprotectant is difficult in order to carry out in vitro cell culture analyses. Freeze-drying can greatly influence nanoparticle size, release behavior, and therefore drug pharmacokinetics [29]. Stirring overtime for solvent evaporation led to very low yields of nanospheres. Hence, to compromise the yield, we attempted centrifugation method by extensive washing of the nanospheres with purified water (milli-Q) and careful separation of the supernatant containing the solvent along with water. Centrifugation has been reported as a more efficient and economic technique for purification of nanoparticles. It also can act to concentrate the nanoparticles in suspension [35]. Preparation/synthesis of nanoparticles by o/w emulsion and solvent evaporation method has been reported to result in larger nanoparticles as compared to those prepared by nanoprecipitation method, and larger nanoparticles have been suggested to be more effective for local delivery than systemic drugs [32]. We have used 1% (w/v) hydroxypropyl methyl cellulose as a mild emulsifier that has excellent implications as a non-toxic excipient in pharmaceutical formulations suitable for improved drug release and increase in the bioavailability of poorly soluble drugs [36]. The amount of emulsifier is critical for nanospheres formation, yield and aggregation tendency. Low amounts of emulsifier lead to aggregation and little recovery of nanoparticles, while too much emulsifier concentrations can lead to reduced drug incorporation [29]. Further, sonication of the polymers in solvent with the drug resulted in lower yield and varied drug loading capacity. Hence sonication prior to addition to external aqueous phase was not adopted. Thereby, we have developed a facile protocol for preparation of PCL/PEG polymeric nanospheres with stable incorporation of AcNDGA to a yield of 89.41% (Table 1).

**Table 1:** Drug load characteristics and zeta potential measurements of PPNS and PPDNS

Nanosphere	Yield (%)	Drug load (mg)^a^	Drug loading capacity (%)^a^	Encapsulation efficiency (%)^a^	Zeta potential (mV)
PPNS	72.86	–	–	–	− 10.34
PPDNS	89.41	13.96	46.53	69.8	− 0.45

^a^PPDNS in Acetonitrile

### Drug loading capacity, Encapsulation efficiency and in vitro drug release

The drug loading capacity and encapsulation efficiency of PCL/PEG/AcNDGA nanospheres in acetonitrile is shown in Table 1. The drug-incorporated polymeric nanospheres exhibited a drug load of 10.0 ± 0.5 µg drug per mg of nanospheres with 49.95 ± 10% encapsulation efficiency and 33-41% drug loading capacity with different batches of nanospheres preparation. A 20 mg of drug was feeded during preparation of the nanospheres. Lower amount of drug (6 mg) resulted in poor drug incorporation and complete drug release due to erosion from the polymer matrix. It has been reported by Li et al. [32] that a suitable amount of feeding drug is required to obtain maximum drug loading capacity and encapsulation efficiency. However, too much drug feed can diminish drug loading content by reflecting largely on the stability of the nanoparticles after reaching saturation solubility in the polymer matrix [37].

**Fig. 3:**
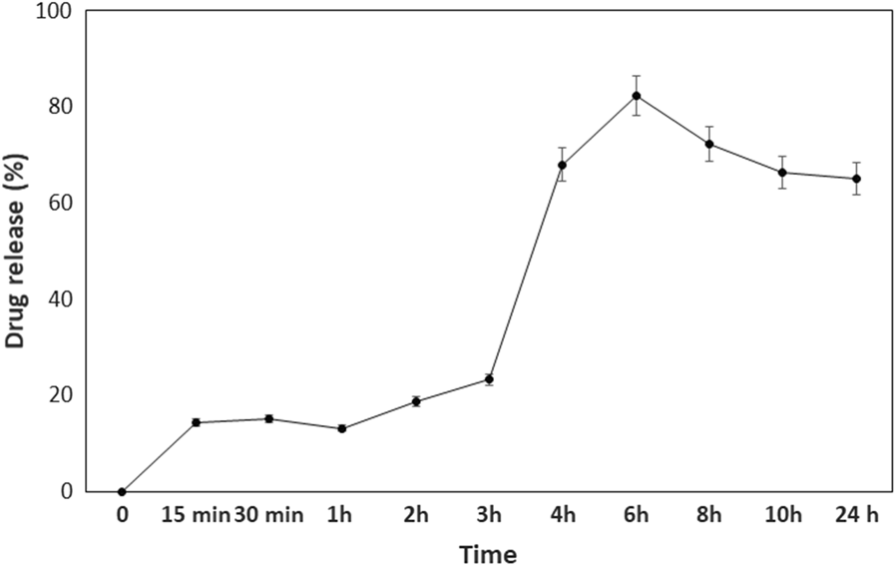
In vitro drug release profile of PPDNS in Methanol

The drug release profile of PPDNS in methanol is shown in Fig. 3. A gradual increase in drug release was observed between 15 min to 2 h and an initial burst release of the drug was obtained by 3 h which further increased linearly up to 6 h. Maximum drug release of 82% (220.64 µg) was obtained at 6 h. A lower release profile was observed after 6 h instead of remaining almost constant that should be achievable after maximal release. This could be due to small amounts of reverse diffusion of drug into the polymer matrix during temperature variation. The initial burst release is attributed to the release of the drug from the surface of the nanospheres [32]. Large particles have low initial burst release and exhibit a longer sustained release pattern, while higher drug loads lead to increased burst release and fast release rates [29]. The release pattern is not in a controlled fashion but is sustained over a period of time. Thereby, we have encountered drug release by erosion from polymer matrix due to low drug feed, release from surface of nanospheres causing initial burst release and by diffusion from the polymer matrix as discussed by Dadwal [16]. The gradual decrease upon longer hours of incubation observed in our study could be due to the incubation of the samples in the Rotaspin instrument at 4 °C with constant agitation to avoid heat generation at room temperature over a period of 8–24 h. The decrease in the drug amount that is released at 8 h, 10 h and 24 h were of the order of 10%, 16% and 15% respectively which is not appreciably significant. Thereby, we have observed a temperature-dependent decrease in drug release profile at 4 °C.

We have used a solvent system for estimation of drug loading content and for release profile due to the hydrophobicity of the drug in phosphate-buffered saline (PBS). Also, the quantification procedure of the drug is by UV-spectroscopy in acetonitrile or methanol at 282 nm or 284 nm respectively [28]. We have used acetonitrile for determining drug load as the drug was completely soluble in acetonitrile which can give a direct measure of the drug that has been incorporated in the polymeric nanospheres as all the drug that has been taken up by the polymer would be present in acetonitrile [32]. Likewise, in drug release study, methanol has been used for dispersion of the nanospheres and mixing that would facilitate drug release at definite time intervals. Drug release profile in solvent, with acetonitrile has also been reported by Li et al. [32]. This validates our drug release experiment in an organic solvent system. Under physiological conditions, the released drug could be taken up into the cell milieu through receptor-mediated endocytosis or by phagocytosis. Although PBS has not been used for drug release experiments, the solvent system is also viable to evaluate in vitro release study. pH-dependent drug release in PBS has been observed by Nimesh et al. [38].

### Characterization of drug loaded PCL/PEG nanospheres

Characterization of nanoparticles is compulsively necessary to pre-determine the drug interactions with cell surface receptors and the release properties in vivo. There does not exist any standardized procedure laid down for usage of particular techniques to characterize nanoparticles. In fact, there is no FDA approved regulatory protocols to undertake characterization techniques. Nevertheless, characterization of nanoparticles is significant in the evaluation of the drug-carrier properties and its pharmacokinetics. Therefore, the drug loaded nanospheres were characterized extensively by various spectroscopic and microscopic techniques to assess its suitable characteristics as a nanostructured drug complex, and evaluate its potential as a drug candidate for therapeutic delivery.

### Particle size distribution and zeta-potential measurements

**Fig. 4:**
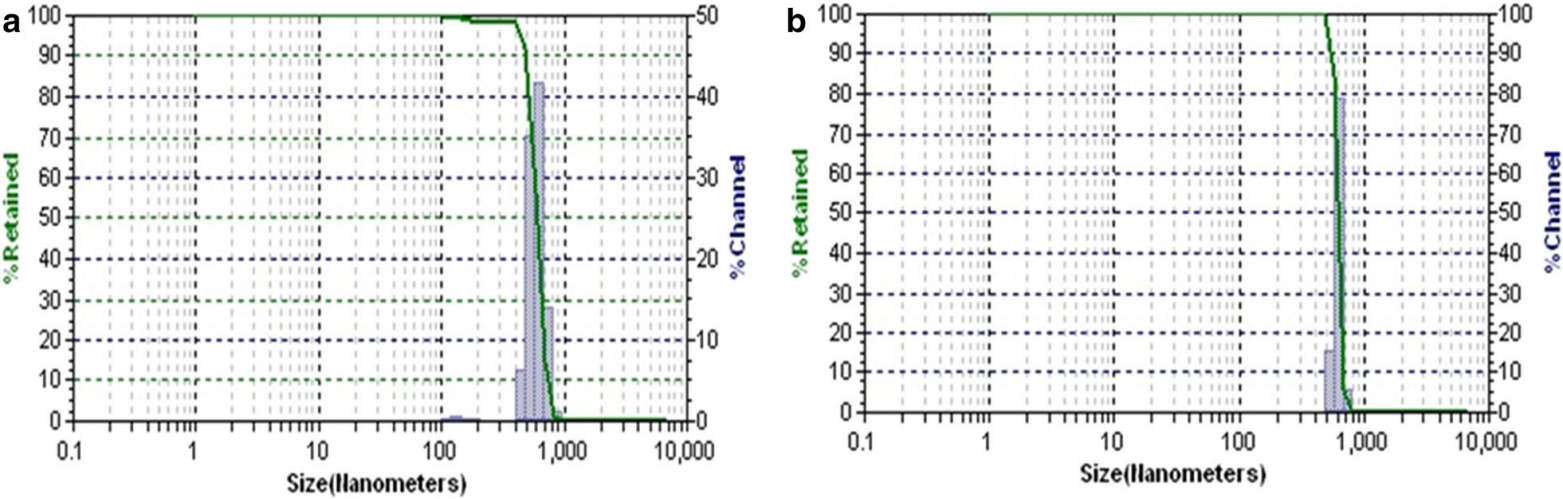
Size distribution of PPNS & PPDNS by Dynamic Light Scattering: **a** PPNS in water; **b** PPDNS in water

The particle size distribution of PPNS and PPDNS was measured by dynamic light scattering technique along with zeta potential measurements. The nanospheres dispersed in water were used as samples. The size distribution of PPNS was between 450 and 750 nm and the size distribution of PPDNS was between 550 and 700 nm exhibiting polydispersity (Fig. 4). Nanoparticles are those materials with sizes between 1 and 100 nm. However, polymeric nanoparticles are an exception with sizes between 10 and 1000 nm which can be explained due to the bulk molecular weights of the polymers used. Other polymeric nanoparticles have also indicated such size range [31–34]. The size parameters are determined by the preparation techniques used to obtain the nanoparticles [39]. The composition of the components including the solvents/surfactants and the process variables during the nanoparticles formation are primary factors that govern the size and morphology parameters. Moreover, it has been proposed by Hickey et al. [40] that improved therapeutic delivery can be achieved by size control of polymeric nanoparticles. Thereby, the size of polymeric nanoparticles is a critical factor while considering the therapeutic potentials of polymeric nanoparticles.

Zeta-potential indicates the charge on the particle surface [41] and the extent of the surface hydrophobicity [16]. The nature and strength of the particle surface charge determine the interactions of the nanoparticles with the surrounding biological environment. Zeta-potential is influenced by the nanoparticle composition and the dispersant medium, and is indicative of the stability of the colloidal suspension [33]. The zeta-potential values can offer predictions about the aggregation tendency of the nanoparticles. Negatively charged nanoparticles are considerably regarded as stable without aggregation in solution. Values close to ± 30 mV represent stable nanoparticle suspensions. However, under practical conditions, zeta-potential values are usually negative and lower [31–33]. In our study, we have obtained negative zeta-potential values with − 10.34 mV for PPNS and − 0.45 mV for PPDNS (Table 1). PPNS is more stable than PPDNS, although this value of PPDNS reflects increased stability of the drug due to steric interactions with PEG [31].

In PBS, aggregation takes place due to ionic interactions of polymeric nanospheres (data not shown). In water, the nanospheres are dispersed by probe sonication and precise size measurements are obtained. The purpose of the dynamic light scattering experiment is only to measure particle size and does not reflect in vivo conditions. Hence water was used for particle size and zeta-potential measurements. Similarly, water has been used for particle size measurements by Li et al. [32] and Campos et al. [33]. Water is a good dispersant medium for particle size measurements as it does not induce aggregation. Hydrophobic drug-loaded nanoparticles need water as dispersant medium to prevent aggregation whereas, for hydrophilic drugs, PBS may be used as it would not induce aggregation. In either case, size measurement by DLS technique is not indicative of behavior of nanoparticles in physiological environment.

### Structural analysis of PPDNS

**Fig. 5:**
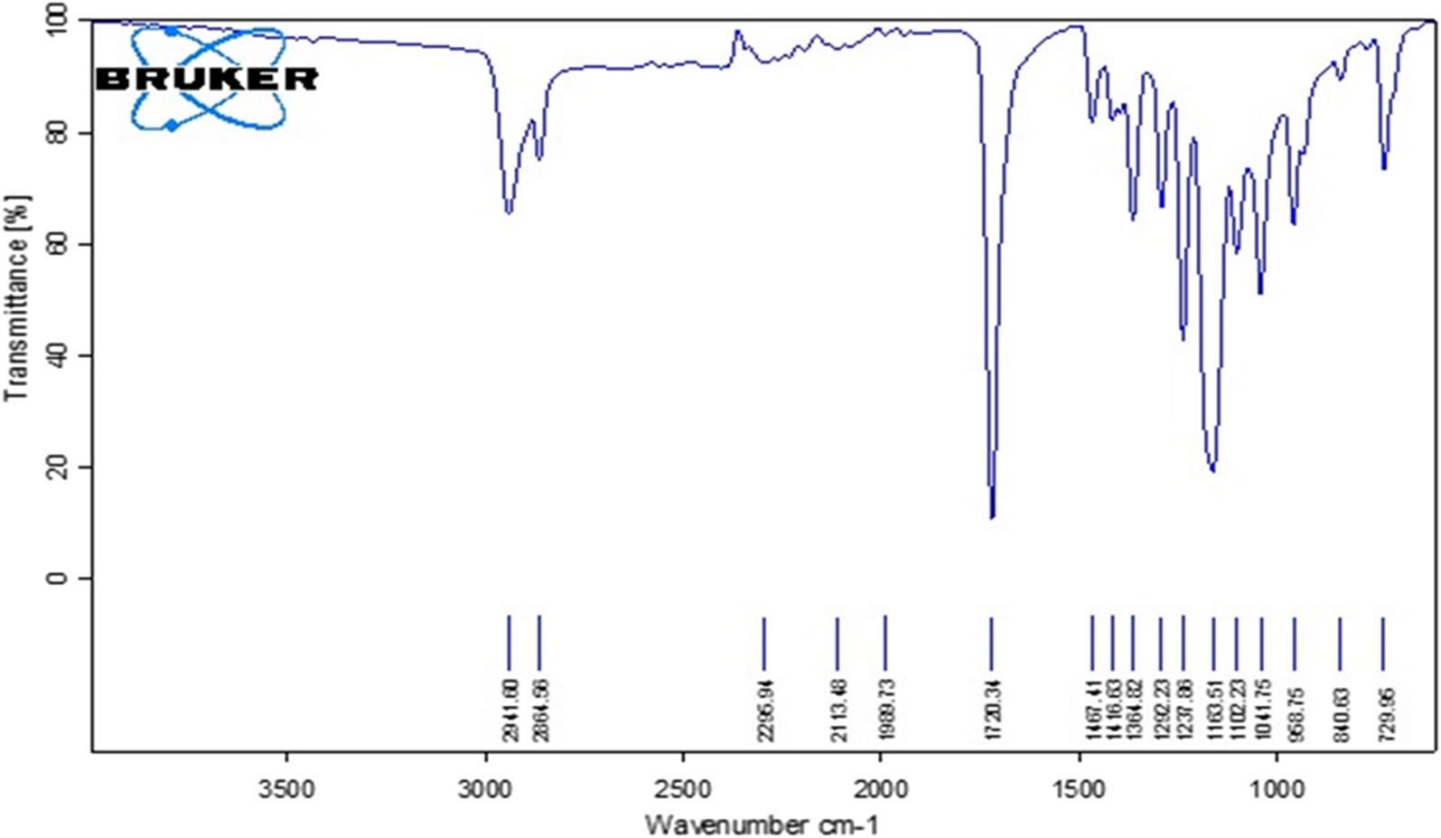
ATR FT-IR spectrum of PPDNS

FT-IR is useful for investigations of structural properties of polymeric nanoparticles and the drug-polymer molecular interactions [42]. ATR FT-IR is being used in recent studies as a sampling technique which can examine the surfaces of materials that may be in solid state or liquid state without further derivatizations with Potassium bromide, as in the case of FT-IR [34]. The ATR FT-IR spectrum of PPDNS prepared by mechanical stirrer method is shown in Fig. 5. PPDNS showed similar bond stretching for their functional groups, indicating intact polymer matrix. There was a strong alkane C–H stretch at 2941–2864 cm^−1^, a strong carbonyl C=O stretch at 1720 cm^−1^ and a variable alkane C–H bending at 1467–1365 cm^−1^. The ether C-O stretch was strong between 1163 and 1041 cm^−1^ and the characteristic fingerprint region was observed between 959 and 730 cm^−1^. The bond stretching in AcNDGA was masked as there was no ester C=O stretch in PPDNS suggesting inaccessible ester linkage.

**Fig. 6:**
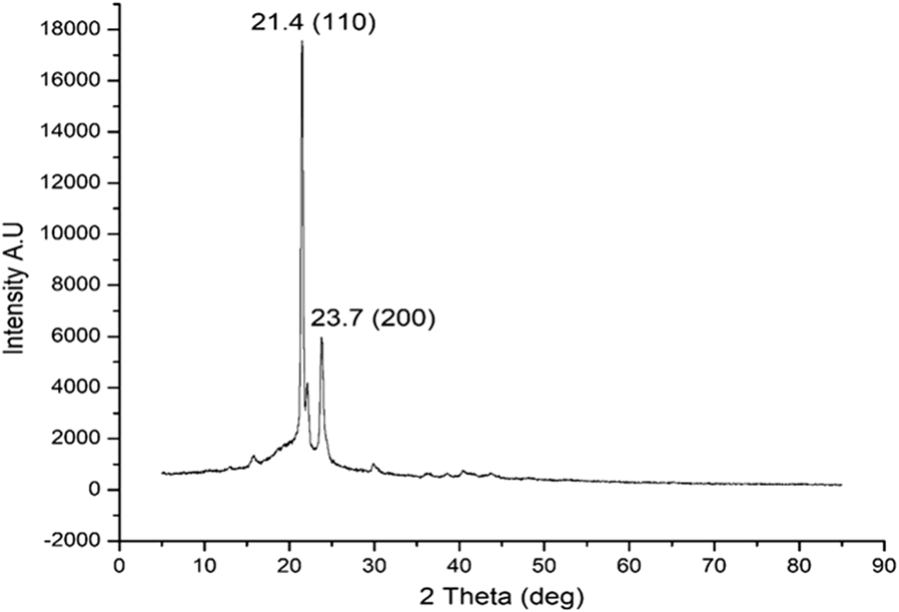
Powder XRD spectrum of PPDNS

Powder X-ray spectroscopy technique provides an X-ray diffraction pattern characteristic of the compound in a powder/amorphous state of the sample in contrast to X-ray crystallography where the technique can analyze only the crystalline lattices of a crystal specimen. For most materials it is very difficult to obtain 100% pure crystal structure which is a drawback of this technique. Therefore with powder XRD, it is possible to analyze various samples irrespective of their physical state. The powder XRD pattern of PPDNS is shown in Fig. 6. The spectrum indicates that the semi-crystalline lattice structure of the drug-incorporated nanospheres with peak intensities corresponding to PEG polymer. However, the nanospheres appear in powder state due to the blend of PCL/PEG. The diffraction pattern is characteristic of the PPDNS sample.

**Fig. 7:**
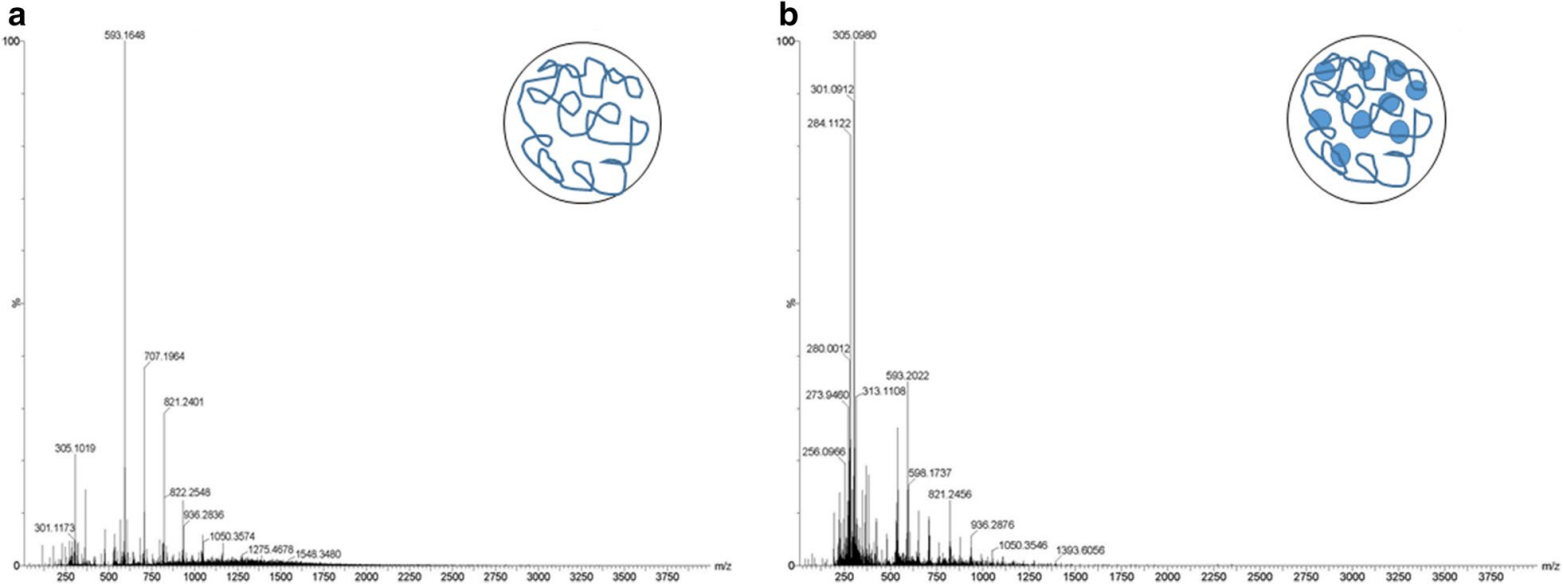
ESI-MS spectrum of: **a** PPNS; **b** PPDNS

ESI–MS is a promising tool not only to investigate the molecular mass of a sample but it also complements circular dichroism spectroscopy to investigate protein folding and further to study post-translational modifications [43]. The mass spectrum of PPNS showed a single peak with molecular mass of 593.16 with sub-populations of 707.19, 821.24 and 305.14 due to the polymeric nanospheres. Generally, it is difficult to predict the exact molecular mass of the polymers due to variations in their molecular structure and sub-populations are obtained. However, in the case of drug-loaded nanospheres, increased molecular masses of several subpopulations are obtained indicating the random association of the drug with the polymer matrix. This random association could be due to the surface and interior matrix binding of the drug with the polymer confirming the nanosphere formulation. Thereby, ESI–MS seems to be a versatile technique to study association of drugs within the polymeric matrix structure providing an analysis of nanosphere structure distinct with nanocapsule formulation (Fig. 7).

### Microscopic analysis of morphology, size and elemental composition

**Fig. 8:**
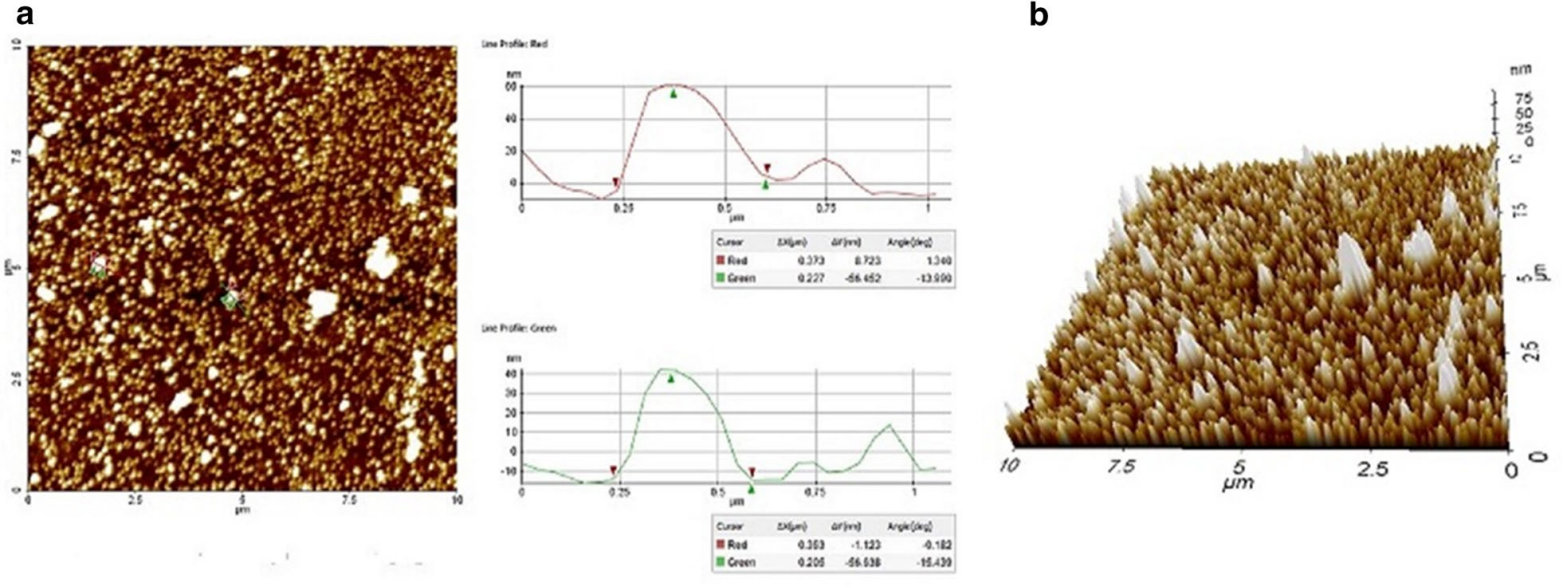
AFM analysis of PPDNS: **a** 2D image; **b** 3D scan at nanoscale

AFM is a technique to monitor the force exerted between the nanomaterial surface and the probe tip. This technique provides an exact and accurate analysis of size, shape, size distribution and aggregation of the polymeric nanoparticles. AFM technique replaces the conventional Transmission Electron Microscopy (TEM) used to estimate particle size and shape and was considered as the ‘gold standard’ to characterize nanoparticles. The sample preparation is relatively simple and requires only purified water as the aqueous phase to disperse the nanomaterials followed by evaporation. However, careful sample preparation is required as it influences the concentration and aggregation, and therefore the size during analysis. Proper evaporation is necessary to ensure aggregation due to coalescence caused by hydrophilicity is avoided [44]. It is also feasible that aggregation may tend to occur during drying process which can be observed in AFM images as larger composites [45]. AFM is being considered as a recent and reliable technique for biomedical applications [46]. The 2D and 3D scans of PPDNS analyzed by AFM are shown in Fig. 8. The results indicate mean sizes of nanospheres between 40 and 60 nm as compared to the apparent size range of 500–700 nm obtained by DLS technique. This apparent size range or the hydrodynamic particle diameter by DLS is due to the formation of hydrodynamic layers surrounding the nanoparticles due to hydrophilicity leading to over-estimation of particle size, whereas in AFM, dehydration of sample occurs prior to analysis [47], providing actual information of size parameters.

**Fig. 9:**
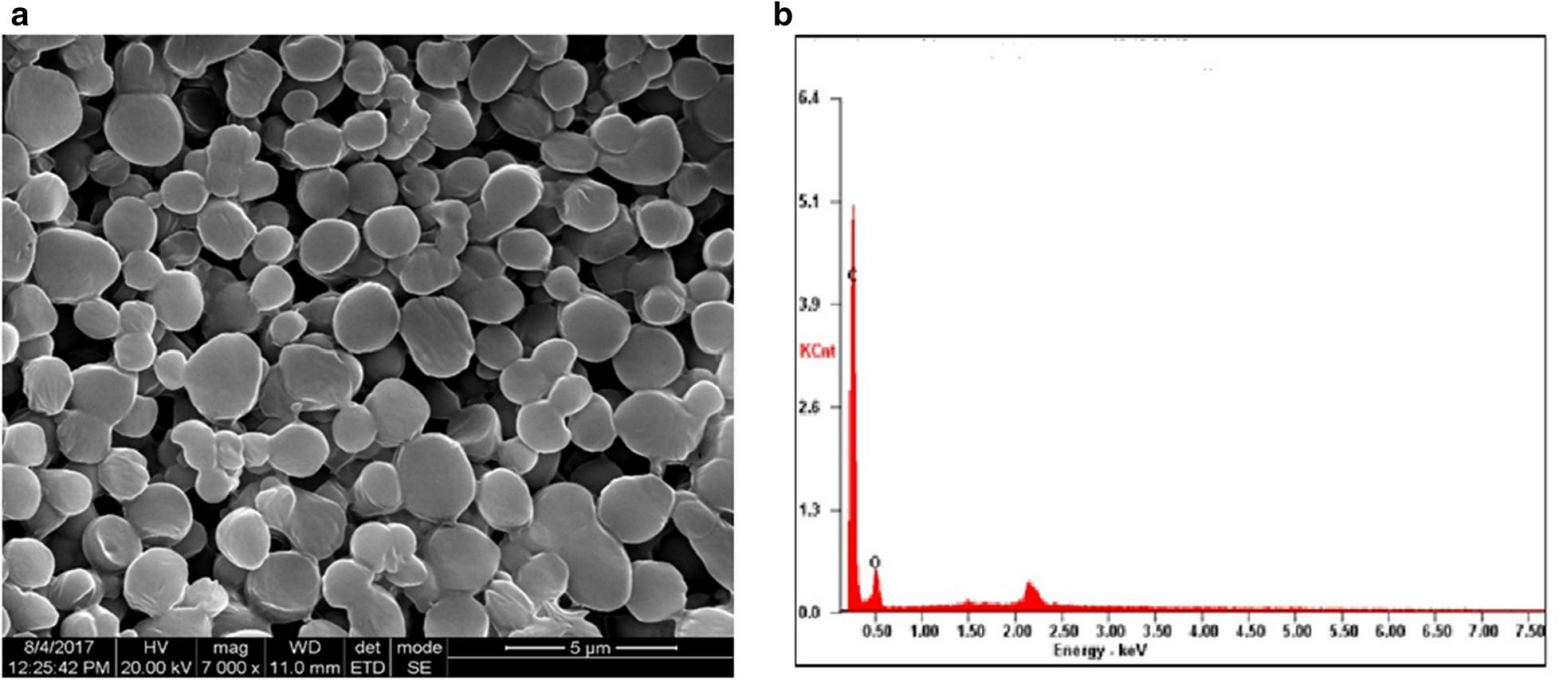
HR-SEM image of PPDNS (**a**) and corresponding EDX spectrum of PPDNS (**b**)

SEM is often a preferred technique to study surface features of materials in the nano and micro ranges. For studies of nanoparticles, HR-SEM provides valuable information on the size, size distribution and the surface morphology of polymeric nanoparticles with additional information of the purity and degree of aggregation of the samples [42]. Combined with SEM, EDX can analyze the elemental composition of the materials when the sample is bombarded by an electron beam. The materials emit X-rays characteristic of the element present providing a ‘fingerprint’ identification of the element. The results of HR-SEM analysis of PPDNS and EDX spectrum are shown in Fig. 9. The SEM images indicate the spherical surface morphology of PPDNS. There were also no pits in the nanospheres due to HPMC external phase [48]. PPDNS nanospheres exhibited polydispersity (Fig. 9a). The EDX spectrum reflects the chemical composition of the samples by displaying peaks corresponding to the energy levels from which X-rays are released by the samples. Each peak is specific for a single element and the higher the peak, the more concentrated the element exists in the sample. In Fig. 9b, PPDNS shows the presence of C-atom and O-atom in the polymeric nanospheres. H-atoms are not obtained as a peak due to lower signal of X-rays released from its electron. EDX spectra thereby cannot detect H-atoms [49].

### Physicochemical characteristics of PPDNS

**Fig. 10:**
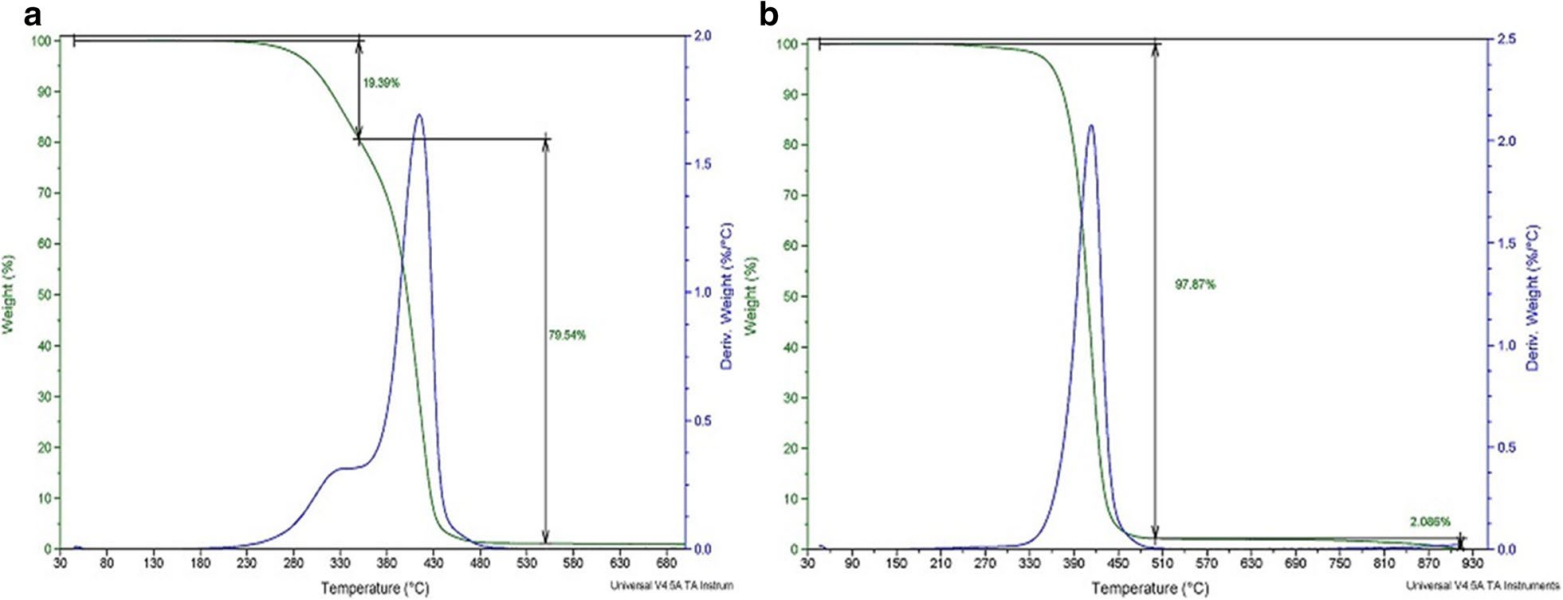
TGA curves of: **a** PPNS; **b** PPDNS at a constant heating rate of 20 °C/min

Thermogravimetric analysis is a useful technique to determine the amount of conjugation of the drug in the nanoparticle. The change in composition of nanoparticles is reflected by a change in temperature weight loss [50]. The thermal degradation behavior of PPNS and PPDNS by TGA analysis is shown in Fig. 10. PPNS exhibited 19.3% weight loss at 350 °C and complete weight loss at 550 °C. The decomposition temperature of PPDNS was lowered to 500 °C with complete weight loss, around 97.87% of the sample. This change in weight loss of PPNS and PPDNS is attributed to the incorporation of the drug in the polymer matrices, further indicating lower interaction of the drug molecule with the polymer to allow for efficient drug release.

**Fig. 11:**
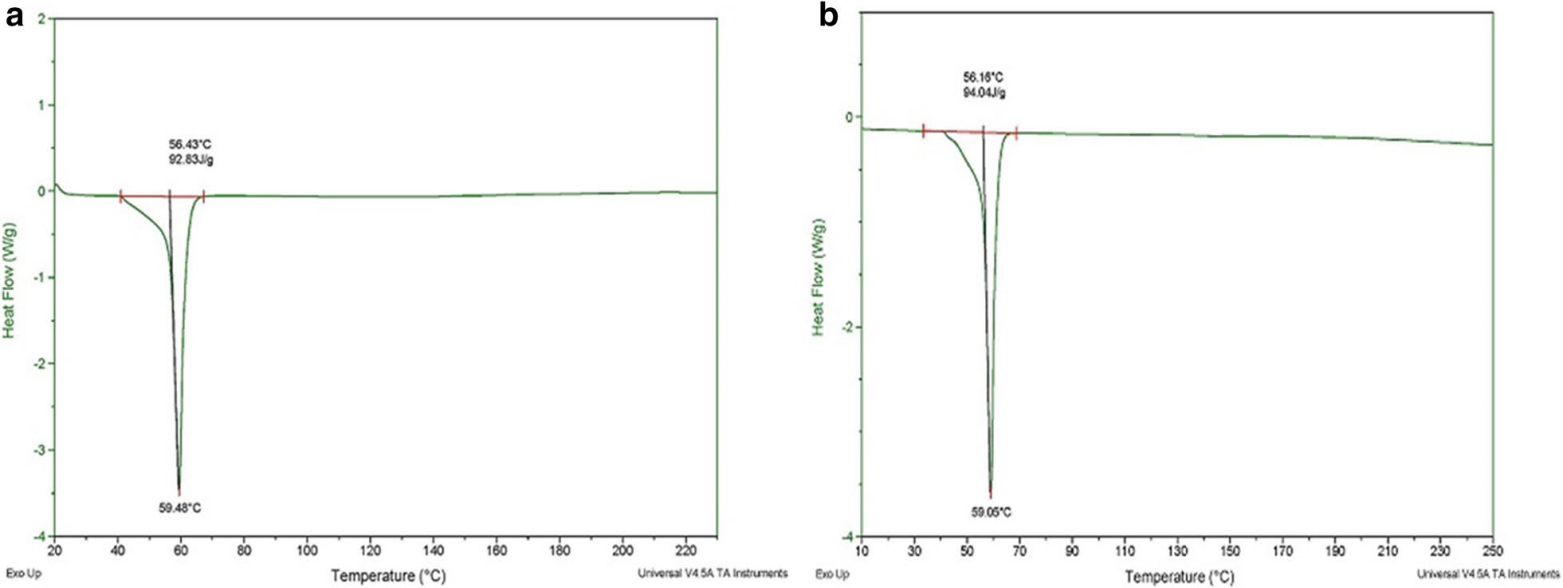
DSC graphs of: **a** PPNS; **b** PPDNS at a constant heating rate of 20 °C/min

DSC analysis can be used to determine the structure, stability and conformation of nanomaterials. The physical transition states of the nanomaterials can also be studied with this technique. The glass transition and melting temperatures of nanomaterials can also be measured [35]. The DSC curves of PPNS and PPDNS at a constant heating rate of 20 °C/min are shown in Fig. 11. Upon heating the polymeric nanospheres at a constant heating rate, a definite amount of heat is transferred to the sample, and the sample temperature reaches a certain amount. The amount of heat it takes to bring about a certain temperature increase is known as the Heat capacity (C_p_) which can be calculated from the DSC plot. The C_p_ can be calculated from the heat flow and heating rate.

In the case of PPNS,

C_p_ = 92.83 J/g/20 °C = 4.64

and for PPDNS,

C_p_ = 94.04 J/g/20 °C = 4.70

Upon further heating, the DSC plot shifted downwards due to the change in heat capacity at its glass transition temperature. The glass transition temperatures of PPNS and PPDNS were 56.43 °C and 56.16 °C respectively. However, the DSC plot did not reflect any increase in heat capacity and the glass transition occur rapidly indicating rapid crystallization. After glass transition the molecules become increasingly mobile and another thermal transition occur known as melting. The melting temperatures (T_m_) of PPNS and PPDNS were 59.48 °C and 59.05 °C respectively. However, the crystallization transition was very rapid and did not show crystallization peak due to the nearly amorphous nature of the nanospheres. The DSC plots of PPNS and PPDNS were similar indicating that the incorporation of drug did not affect polymer matrix as well as crystallization and melting states of polymers.

In vitro cell inhibition by MTS assay with HepG2 liver cancer cells

**Fig. 12:**
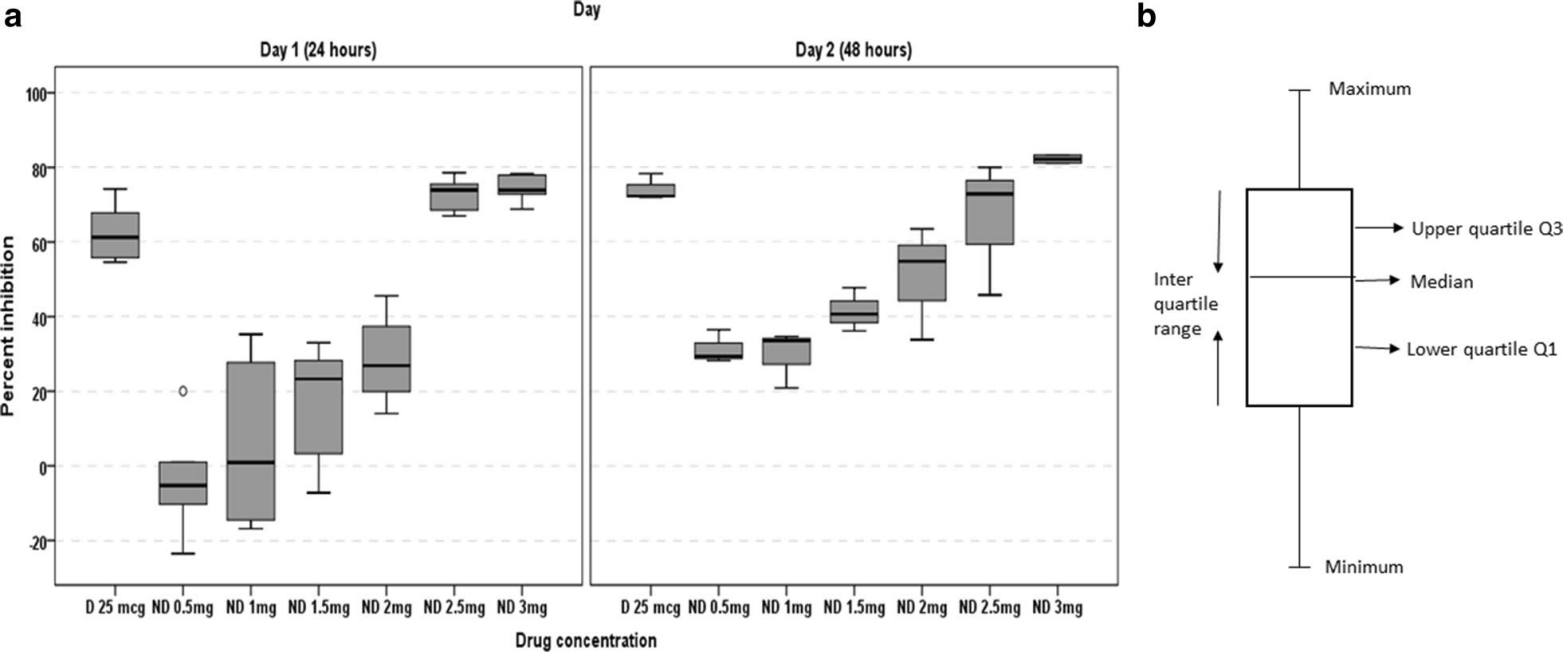
**a** Cytotoxicity assay of HepG2 cells treated with free AcNDGA (D) and PPDNS (ND) with MTS Reagent at 24 h (Day 1) and 48 h (Day 2). Maximum cell inhibition is observed at 24 h with 2.5 mg PPDNS containing 25 µg AcNDGA as against 25 µg of free AcNDGA at 48 h. *P*-value is < 0.05 on Day 1. *P*-value on Day 2 is N.S. **b** Representation of a Box-plot

Primary liver cancer is globally the sixth most frequent cancer (6%) and the second leading cause of death from cancer (9%). In 2018, it occurred in 841,000 people and resulted in 782,000 deaths. In 2015, 263,000 deaths from liver cancer were due to hepatitis B, 245,000 to alcohol and 167,000 to hepatitis C (*Source*-Wikipedia, accessed 7th January 2020). The Indian subcontinent is facing a serious threat due to HCC arising secondary to viral hepatitis infection, Hepatitis B or Hepatitis C with a mean incidence of 4.15%. In context of high incidences of Hepatitis infections in India with over 3–9 million as of 2018, and liver cancers arising secondarily to cirrhosis and alcoholism as well, it is vital to develop new and efficient drugs for therapeutic strategies to treat liver cancer. Considering these factors, we have tested the Acetyl NDGA Nanoformulation for its cytotoxicity to liver cancer cells, HepG2, by a one-solution MTS assay. The MTS assay results for percentage inhibition of HepG2 cells by AcNDGA and AcNDGA/PCL/PEG (PPDNS) nanospheres are shown in Fig. 12 as a box plot graph. Box-plot and the median test results along with *P*-values are generated by SPSS v.16.0 (Table 2). As the sample size is small, non-parametric test has been applied and any *P*-value less than 0.05 is considered as statistically significant.

**Table 2:** Median, minimum, maximum and *P*-values of comparison between free AcNDGA and PPDNS (drug-loaded polymeric nanospheres) at different concentrations from ANOVA statistical analysis using SPSS v.16

	Day 1 (24 h)				Day 2 (48 h)			
Drug	Median	Minimum	Maximum	*P*-value	Median	Minimum	Maximum	*P*-value
D 25 µg	61.27	54.61	74.21	Standard	72.31	72.06	78.35	Standard
ND 0.5 mg	− 5.29	− 23.53	20.03	0.002	29.35	28.23	36.50	0.10
ND 1.0 mg	0.89	− 16.86	35.27	0.002	33.57	20.87	34.67	0.10
ND 1.5 mg	23.30	− 7.25	32.98	0.002	40.65	36.19	47.74	0.10
ND 2.0 mg	26.88	14.00	45.55	0.002	54.81	33.79	63.50	0.10
ND 2.5 mg	73.93	67.01	78.56	0.026	72.91	45.81	80.05	1.00
ND 3.0 mg	73.92	68.80	78.36	0.015	82.22	81.16	83.29	0.20

D-Free AcNDGA; ND-PPDNS. Drug load of PPDNS (ND) = 10 µg drug per mg nanospheres

*P*-value less than 0.05 is considered as statistically significant

Day 1 *P*-values were *p ≤ 0.05. Day 2 *P*-values were not significant (N.S.)

The graph indicates maximum percent inhibition with free AcNDGA (D) at 25 µg on Day 2 and PPDNS (ND) at 2.5 mg (containing 25 µg drug) on Day 1 with median values of 72.31% and 73.93% respectively. The whiskers of box plots of D 25 µg and ND 2.5 mg suggest minimal variation of data on the positive side of the interquartile groups and were similar in the lower quartile group. The medians also appear similar with ND 2.5 mg (73.93%) and ND 3.0 mg (73.92%) on Day 1. The box plots of ND 0.5 mg, 1.0 mg, 1.5 mg and ND 2.0 mg all exhibited very low percent inhibition values on Day 1 but were significantly higher on Day 2 with comparatively short box plots exhibiting similarity in each group.

These data conclude that the drug-loaded nanospheres at 25 µg exerted early cell death on day 1, which is appealing to the use of the nanoformulation than the free drug. These results suggest the rapid uptake and targeted release of the drug to induce early cell death. Although increased cell death occurred with 3.0 mg of PPDNS on day 2, it can be speculated that increasing drug concentrations can also promote drug-induced toxicity especially with longer drug treatment time periods and also the difference is statistically insignificant. Further, we presume that the high cytotoxicity with 3.0 mg of PPDNS on day 1 as well as day 2 could be due to necrotic cell death and does not provide a picture of cytotoxicity. Hence, we consider that 2.5 mg PPDNS containing 25 µg AcNDGA was effective in inducing HepG2 cell death on day 1. It is also to be considered that controlled drug release is desired for an effective drug delivery strategy and the role of nanoparticles in drug delivery emphasizes such an advantage of drug release. In a report by Myint et al. [51], they have observed maximum cytotoxicity at 24 h at a concentration of 100 µg/ml of the drug extract to HepG2 cells, while in our study, 25 µg concentration was sufficient to induce about maximum cell death. This result is in favor of the fact that the therapeutic dosage should be at a minimal range to avoid drug-induced toxicity and its side effects. In a recent review, cell-based assays such as cytotoxicity or anti-proliferative assays in vitro have been considered as viable protocols for evaluation of anti-cancer potential [52]. In this regard, the drug-incorporated nanospheres are ideal candidates than their free counterpart for drug delivery to human hepatoma HepG2 cells.

Therefore, the Acetylated NDGA derivative as a nanoformulation seems to possess effective anti-cancer potential which can be further elucidated by in vitro and in vivo experiments for desired therapeutic and pharmacological applications. In this context, we have evaluated that NDGA derivatives are better alternatives for drug therapy as we have suggested in our recent article by Mala et al. [53].

## Conclusions

NDGA finds a place in traditional medicinal practice for remedy of multiple medical disorders, and is consumed as ‘chaparral’ tea infusions in North America and Mexico. Few reports have indicated hepatotoxicity and formation of renal cysts with prolonged use. There have been recent interests to synthesize NDGA derivatives by chemical methods and evaluate their bioactivities. Previously, a tetra-acetylated NDGA derivative has been synthesized and evaluated for anti-cancer activity in adenocarcinoma and melanoma, and in combination therapy with doxorubicin. We have synthesized the AcNDGA based on this procedure and have prepared a nanoformulation with PCL/PEG by o/w emulsion solvent evaporation method by a simple strategy. The drug loading capacity and encapsulation efficiency of the drug-loaded nanospheres were determined. Further, the nanospheres have been extensively characterized by an array of spectroscopic and microscopic techniques which provided valuable information regarding nano-size regime, molecular interactions between drug and polymer matrix and the release characteristics. The nanospheres exhibited nanoscale features to affect therapeutic delivery to human liver cancer cells, HepG2. Thereby, we have developed a drug delivery module with suitable characteristics and features with therapeutic potential particularly for anticancer treatment.

## Data Availability

All the data pertinent to this work has been submitted here
